# A pneumatic random-access memory for controlling soft robots

**DOI:** 10.1371/journal.pone.0254524

**Published:** 2021-07-16

**Authors:** Shane Hoang, Konstantinos Karydis, Philip Brisk, William H. Grover

**Affiliations:** 1 Department of Bioengineering, University of California, Riverside, CA, United States of America; 2 Department of Electrical and Computer Engineering, University of California, Riverside, CA, United States of America; 3 Department of Computer Science and Engineering, University of California, Riverside, CA, United States of America; Istituto Italiano di Tecnologia Center for Micro BioRobotics, ITALY

## Abstract

Pneumatically-actuated soft robots have advantages over traditional rigid robots in many applications. In particular, their flexible bodies and gentle air-powered movements make them more suitable for use around humans and other objects that could be injured or damaged by traditional robots. However, existing systems for controlling soft robots currently require dedicated electromechanical hardware (usually solenoid valves) to maintain the actuation state (expanded or contracted) of each independent actuator. When combined with power, computation, and sensing components, this control hardware adds considerable cost, size, and power demands to the robot, thereby limiting the feasibility of soft robots in many important application areas. In this work, we introduce a pneumatic memory that uses air (not electricity) to set and maintain the states of large numbers of soft robotic actuators without dedicated electromechanical hardware. These pneumatic logic circuits use normally-closed microfluidic valves as transistor-like elements; this enables our circuits to support more complex computational functions than those built from normally-open valves. We demonstrate an eight-bit nonvolatile random-access pneumatic memory (RAM) that can maintain the states of multiple actuators, control both individual actuators and multiple actuators simultaneously using a pneumatic version of time division multiplexing (TDM), and set actuators to any intermediate position using a pneumatic version of analog-to-digital conversion. We perform proof-of-concept experimental testing of our pneumatic RAM by using it to control soft robotic hands playing individual notes, chords, and songs on a piano keyboard. By dramatically reducing the amount of hardware required to control multiple independent actuators in pneumatic soft robots, our pneumatic RAM can accelerate the spread of soft robotic technologies to a wide range of important application areas.

## Introduction

Pneumatically-actuated soft robots demonstrate certain advantages over rigid robots in many applications. For example, their soft rubbery bodies are suitable for use as robotic grippers for lifting delicate objects [1–3]. Their ability to yield increases their safety in close proximity to humans [4]. Soft robots are also suitable for use *in contact* with humans, as wearable exoskeletons for assisting laborers, warfighters, the elderly, or patients with musculoskeletal or neurological conditions [5, 6]. Additionally, soft robots resemble living organisms more closely, which makes them suitable for use in biomimetics [7–13].

However, existing systems for *controlling* soft robots have significant disadvantages. Many soft robots move by using air under pressure or vacuum to expand or contract flexible actuators inside the robot. The flow of air to each independent actuator is usually controlled by a dedicated electromechanical solenoid valve, which in turn is controlled by an electronic or electromechanical relay, which in turn is controlled by an electronic microcontroller or computer. Ironically, all of this electronic and electromechanical hardware exists to control a robot that is, after all, fundamentally *pneumatic*, not electrical. This mishmash of two very dissimilar domains—pneumatics and electronics—makes current soft robotic systems unnecessarily complex, expensive, bulky, and power-hungry. And while some research has blurred the line between these dissimilar domains using *e.g*. electrically conductive and insulating fluids [14], these approaches still require electronic components and limit the use of these robots in potentially hazardous environments where these components could spark and cause a fire or explosion. Finally, electronic components also hinder the use of soft robots in wearable exoskeletons, therapeutic devices, and other applications in close proximity to humans where lugging around heavy batteries, valves, computers, and other electronics is impractical.

To solve this problem, researchers have turned to an idea that predates electronic computers: *pneumatic logic* [15]. In pneumatic logic, air (not electricity) flows through circuits of tubes or channels, and air pressure is used to represent a logical state (on or off, TRUE or FALSE, etc.). In the decades before electronic logic became ubiquitous, principles of pneumatic logic provided advanced levels of control in a variety of products, including climate control systems (which used all-pneumatic thermostats, bellows, valves, and other components to control temperature and humidity throughout a building [16]) and player pianos (which used air to read punched-paper “programs” and to control nearly 100 independent keys in the early 1900s [17]). Since pneumatic logic is powerful enough to control buildings and pianos, it should also be capable of controlling soft robots.

However, the existing implementations of pneumatic logic for soft robot control have limitations that complicate their use with robots that contain many independent actuators. For example, the pneumatic logic gates and embedded actuators demonstrated by Preston *et al*. [18, 19] are fabricated one-by-one and connected together manually using tubing; this complicates their large-scale use in complex multi-actuator robots which could require tens or hundreds of logic gates. Other designs link pneumatic actuators together mechanically into pneumatic networks that demonstrate feedback-based oscillations [20, 21]; these are very useful for controlling repetitive operations (like walking gaits in legged robots) but are less suitable for individual actuator control and still require manual fabrication and assembly. Researchers have improved the manufacturability of pneumatic logic circuits by using microfabrication to create pneumatic logic circuits [22], but since these logic circuits use normally-open microfluidic valves [23] as transistor-like elements, they are limited to simpler logic circuits like demultiplexers which can only control one actuator at a time. This is because normally-open valves require a constant applied pressure to seal closed; when this pressure is removed, the valves automatically reopen and vent any trapped pressures. With no way to store a pressure differential inside the device, pneumatic logic circuits built with normally-open valves have no “memory” and cannot maintain the state of one soft robotic actuator while setting another. Finally, while valves with bistable buckling silicone membranes have recently been used as single-bit memories in soft robots [24, 25], the large size and manual fabrication and interconnection of these elements again complicates their large-scale use in complex pneumatic logic circuits.

**Fig 1:**
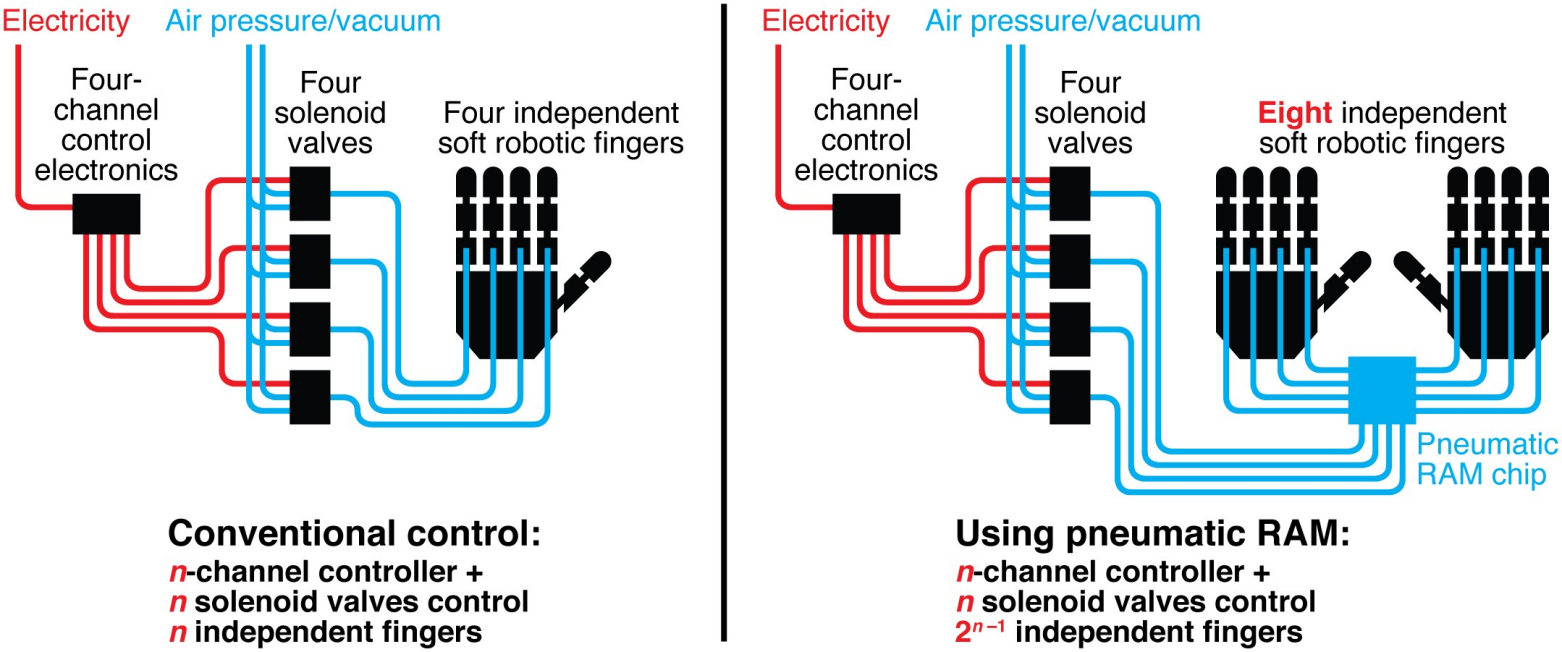
Conventional methods for controlling pneumatic soft robots require a dedicated electronic control line and solenoid valve for each independent actuator (left). Using our pneumatic random access memory (RAM), the same amount of electromechanical control hardware can operate many more actuators while still providing independent control of each actuator (right).

To address these limitations, we developed pneumatic logic circuits *with memory* that can be used to control large numbers of independent soft robotic actuators (Fig 1). We accomplished this by using *normally-closed* microfluidic valves [26] in our pneumatic logic circuits. Since these valves remain sealed against a pressure differential even when disconnected from a pneumatic control line, they can be used to create trapped pressure differentials that function as pneumatic memories and maintain the states of large numbers of soft robotic actuators. And since these valves can be easily fabricated in dense arrays, they support complex circuits that perform advanced operations like time division multiplexing and pneumatic analog-to-digital conversion. These pneumatic logic circuits can significantly reduce the amount of expensive, bulky, and power-consuming electromechanical hardware required to control a pneumatic system.

## Results

### Adapting normally-closed microfluidic valves for controlling soft robots

Our pneumatic memory circuits use a modified form of monolithic membrane valves. These valves were originally developed for controlling fluid flow in microfluidic chips [26] and later used as transistor-like elements in pneumatic logic circuits for controlling microfluidic chips [27–36]. Though perhaps not as well known as the soft lithography microfluidic valves developed by Stephen Quake [23], monolithic membrane valves have several traits that make them particularly useful in pneumatic logic applications. The two valving technologies are compared in Table 1.

**Table 1:** Comparisons between soft lithography valves (used in most previous work on microfabricated pneumatic logic control of soft robots) and monolithic membrane valves (adapted for use in this work).

	Soft lithography valves [23]	Monolithic membrane valves [26]
**Fabrication**:	Channels are cast in silicone rubber by pouring liquid silicone over a positive mold made using photolithography. After curing, the solid silicone channel layers are peeled off the mold. Two or more silicone layers are bonded together; valves are formed wherever two channels cross in different silicone layers.	Channels are etched in rigid glass using photolithography or engraved in rigid plastic using CNC milling. A featureless solid silicone rubber sheet is bonded between two rigid channel layers. Valves are formed wherever an etched/engraved chamber is located across the silicone sheet from a gap in a channel.
**Operation**:	A high pressure expands one channel into a second channel, pinching the second channel closed and closing the valve.	A low pressure in a chamber pulls the silicone sheet away from the gap in the channel, allowing flow across the gap and opening the valve.
**Powered by**:	Pressure	Vacuum
**At-rest state**:	Normally open	Normally closed
**Suitability for use in pneumatic memory for controlling large numbers of independent soft robotic actuators**:	Limited because normally open valves lose control of the contents of the valved channel when disconnected from a pressure source.	Favorable because normally closed valves maintain control of the contents of the valved channel when disconnected from a vacuum source. This enables trapped pressure differences that can serve as “memory” even when the valves are disconnected from power.

**Fig 2:**
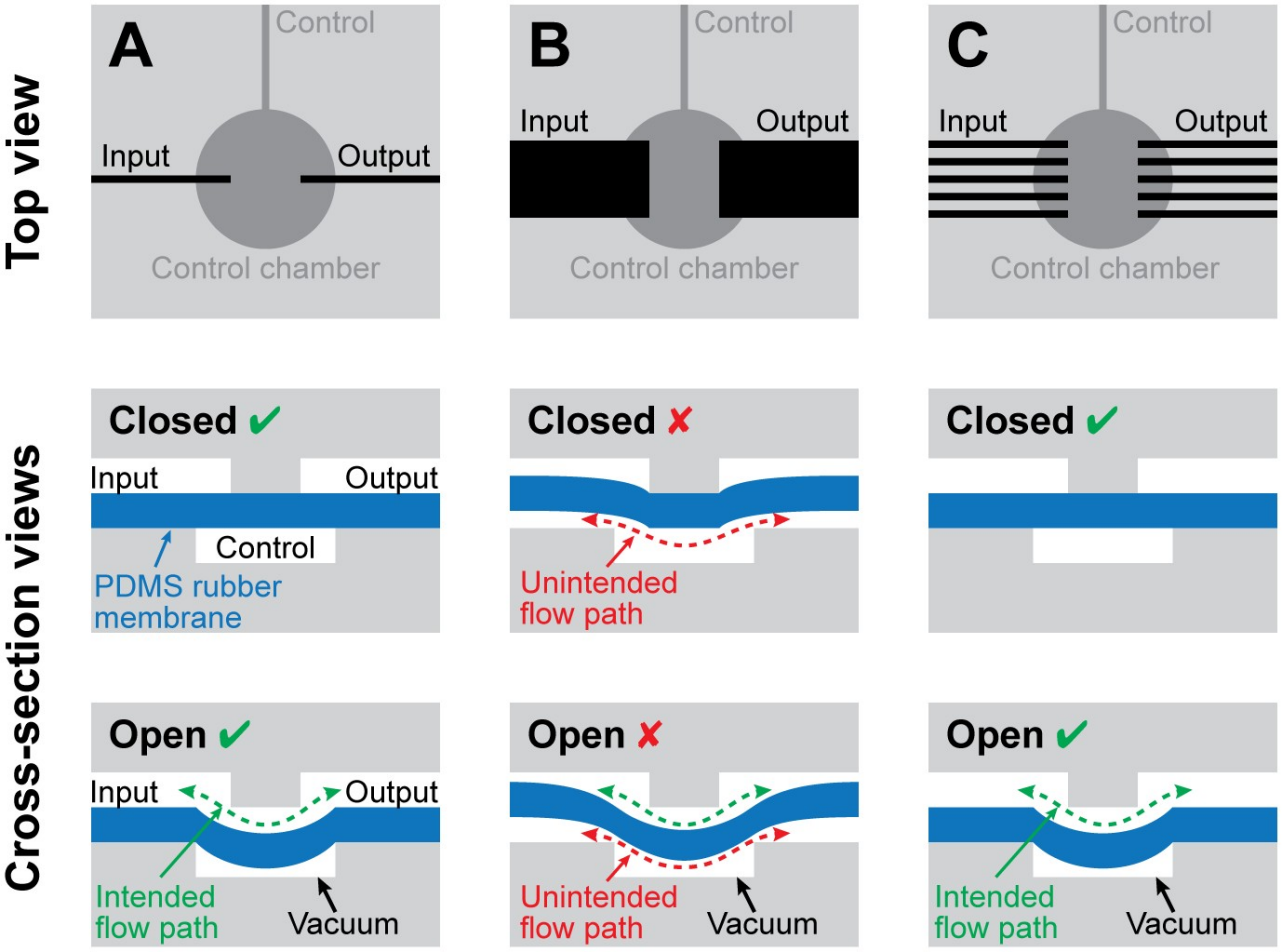
Adapting normally-closed monolithic membrane valves for soft robotic applications. **(A)** In conventional microfluidic monolithic membrane valves [26], fluid flow between the Input channel and the Output channel is blocked by a flexible silicone membrane (blue). When a vacuum is applied to the Control channel, the silicone membrane is pulled into the Control chamber, opening a path for flow between the Input and Output channels (green dashed arrow). Though adequate for microfluidic applications, the rate of air flow through conventional monolithic membrane valves is too small for controlling larger and faster-moving soft robots. **(B)** Increasing the widths of the Input and Output channels significantly increases the rate of flow through the valve, but this change brings an undesirable side effect: the silicone membrane can stretch into the Input and Output channels as if they were Control chambers, creating unintentional flow paths (red dashed arrows). **(C)** Using multiple narrow-width Input and Output channels in parallel eliminates the risk of unintentional flow paths while still maintaining a high flow rate suitable for use controlling soft robotic actuators.

Conventional monolithic membrane valves (Fig 2A) consist of an Input channel and an Output channel in one layer, a Control channel and chamber in a second layer, and a featureless polydimethylsiloxane (PDMS) silicone rubber membrane sandwiched between the two layers [26]. The valve is normally closed, meaning that when the Control chamber is at atmospheric pressure, the PDMS membrane seals against the gap between the Input and Output channels and blocks flow between them. When a vacuum is applied to the Control channel, the PDMS membrane is pulled into the Control chamber and away from the Input and Output channels, thereby opening a path for flow between the Input and Output channels.

Due in large part to their normally-closed nature, monolithic membrane valves have a history of use in complex pneumatic logical circuits for controlling microfluidic devices. For example, the authors and other researchers have demonstrated microfluidic pneumatic versions of Boolean logic gates [27], binary memory [28], mathematical calculators [27], clocks [29], programmable biochemical processors [30–34], and even simple computers (finite state machines [37]) [35, 36].

However, in their conventional form, these pneumatic logic circuits would only be capable of controlling small and slow-moving robots. This is because the small microfluidic-scale channels in these circuits can only accommodate small volumes of air at low flow rates. So while conventional normally-closed valve-based pneumatic logic circuits have been proposed for controlling soft robots [38], experimental demonstrations of these control systems are limited.

To enable monolithic membrane valve-based logic circuits to control larger and faster-moving robots, we first tried increasing the size of the channels in these circuits. As the cross-sectional area of a channel gets larger, its capacity for flow increases dramatically. However, when we tested pneumatic logic circuits with larger channels, we found a fundamental problem with these designs: as shown in Fig 2B, when the widths of the Input and Output channels are increased, the silicone rubber membrane can be pulled or pushed into the Input or Output channels, creating an unintentional new path for air flow on the other side of the membrane (red dashed arrows in Fig 2B). In this manner, the Input and Output channels unintentionally behave like Control chambers, and the pneumatic logic circuit no longer functions as intended.

We then developed a modified pneumatic logic circuit design that handles larger air flow rates without compromising the circuit’s functionality. We accomplished this by using multiple channels in parallel everywhere that high flow is needed. When multiple parallel channels are used as the Input and Output channels in a valve (Fig 2C), the resulting “high-flow” monolithic membrane valve can carry several times more flowing air when open and still function correctly in pneumatic logic circuits. Consequently, high-flow monolithic membrane valves can control larger and faster-moving soft robots than their traditional microfluidic counterparts.

### “Truth table” for high-flow monolithic membrane valves

Next, we needed to define the rules for how high-flow monolithic membrane valves behave in pneumatic logic circuits. While monolithic membrane valves can be thought of as analogous to transistors in electronic circuits, the physics of flowing air in pneumatic logic is fundamentally different from the physics of flowing electrons in electronic logic. In particular, pneumatic logic circuits built from normally-closed valves are capable of storing or trapping pressure differentials inside the circuit; this is fundamentally different from electronic circuits which usually require an additional component (like a capacitor or a floating-gate transistor) to store a charge (the electronic analog of a pressure).

**Fig 3:**
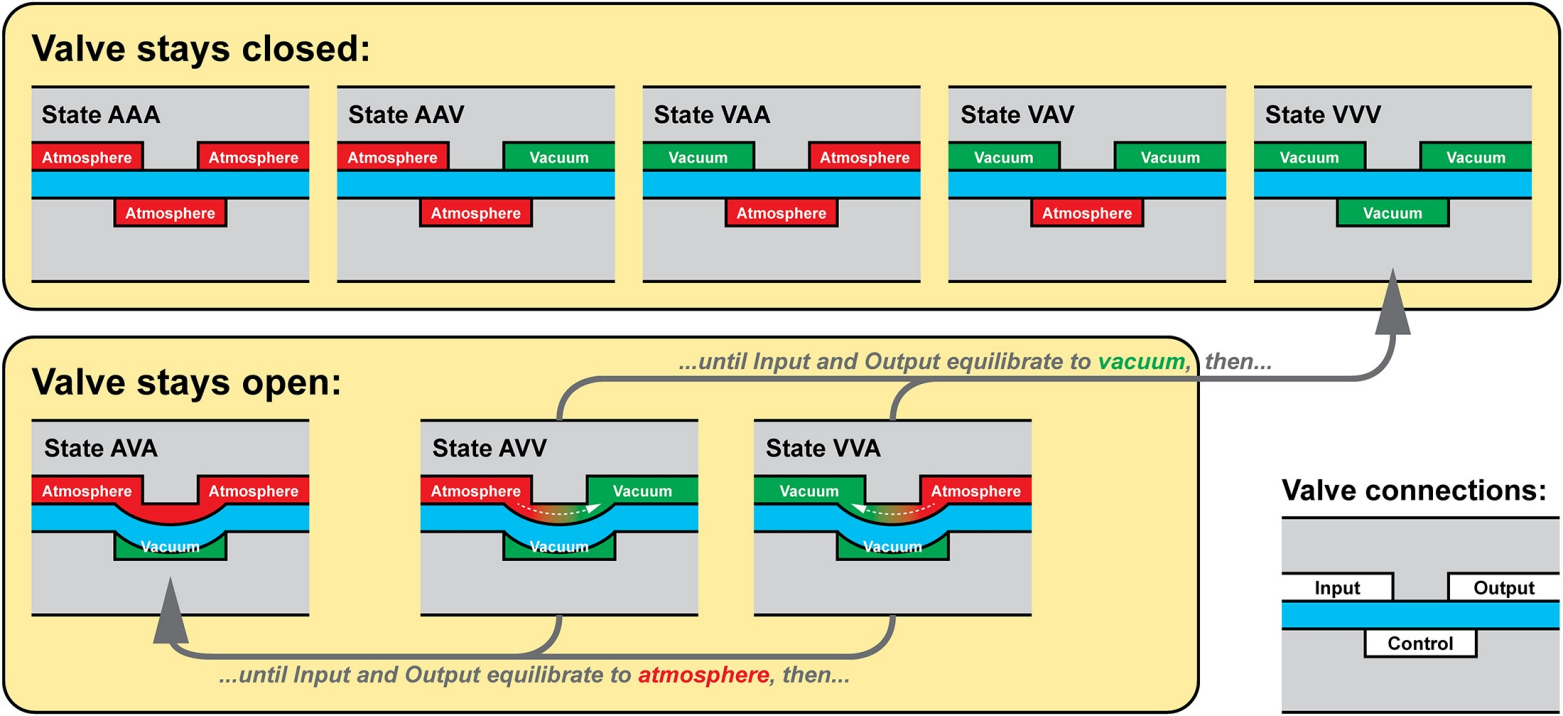
A “truth table” showing cross-sections of a high-flow monolithic membrane valve during each of the eight possible combinations of atmospheric pressure (*A*) and vacuum (*V*) applied to the valve’s three connections (Input, Control, and Output). When atmospheric pressure is applied to all three connections on a valve, the valve has equal pressure applied on both sides of the flexible membrane, so the membrane remains in its resting (closed) state (*AAA*). If the Control connection on a valve receives atmospheric pressure, then the valve will remain closed, regardless of whether the Input and Output connections receive vacuum or pressure (*AAV*, *VAA*, and *VAV*). When vacuum is applied to all three connections on a valve, the valve has the same pressure on both sides of the flexible membrane, so the membrane remains at rest and the valve stays closed (*VVV*). If vacuum is applied to the Control connection while the Input and Output connections are at atmospheric pressure, then the pressure differential across the flexible membrane causes the membrane to stretch into the Control chamber and opens the valve (*AVA*). When vacuum is applied to the Control connection and the Output connection while the Input connection is at atmospheric pressure (*AVV*), the valve opens and air flows from the Input to the Output. Likewise, when vacuum is applied to the Control and Input connections while the Output is at atmospheric pressure (*VVA*), the valve opens and air flows from the Output to the Input. In both cases, the valve remains open and air continues to flow as long as there is a pressure difference between the Input and Output connections. *However, if the pressures at the Input and Output connections equalize (both become vacuum, or both become atmospheric pressure), the valve will automatically transition to a new state*. If both the Input and the Output reach atmospheric pressure, then the valve will transition to state *AVA* and remain open. But if both the Input and the Output reach vacuum, then the valve will transition to state *VVV* and automatically close. This automatic transition from state *AVV* or *VVA* to state *VVV* is particularly useful because it can “trap” a vacuum in a region of a pneumatic logic circuit; the vacuum remains trapped until it is vented by opening a path to atmospheric pressure using state *AVV* or *VVA* again. This serves as a one-bit nonvolatile pneumatic “memory”: a section of channel represents 1 (TRUE) if it contains a trapped vacuum and 0 (FALSE) if it contains atmospheric-pressure air.

A monolithic membrane valve has three connections (Input, Control, and Output, as shown in Fig 2), each of which can receive either vacuum (abbreviated *V*) or atmospheric pressure (abbreviated *A*). This results in eight possible states for the valve (abbreviated in the order *Input Control Output*): *AAA*, *AAV*, *AVA*, *AVV*, *VAA*, *VAV*, *VVA*, and *VVV*. We describe the state of the valve during each of these eight states in the “truth table” shown in Fig 3.

### Design of a pneumatic eight-bit random-access memory

As a proof-of-concept for using high-flow monolithic membrane valves to control soft robots, we designed, fabricated, and tested a pneumatic eight-bit random-access memory (RAM). This pneumatic logic circuit can set and “remember” the air pressure level at eight outputs in the circuit. By connecting soft robotic actuators to these outputs, we can use the pneumatic RAM to control eight independent actuators.

**Fig 4:**
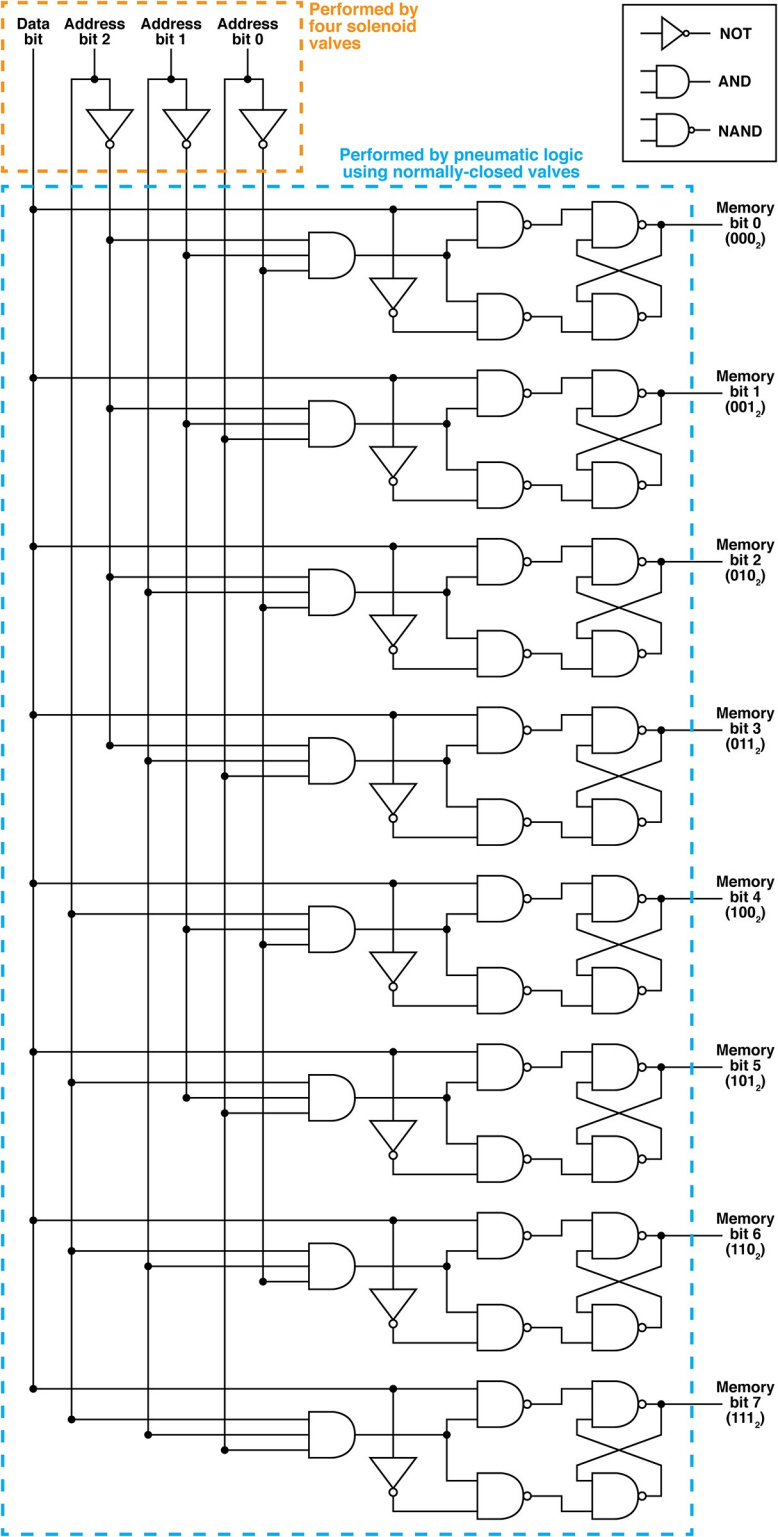
Logic diagram for our pneumatic eight-bit random-access memory. Four solenoid valves (orange region in the diagram) provide three pneumatic Address bits (for selecting which Memory bit to set) and one Data bit (for setting the value of the selected Memory bit); the rest of the logic diagram (blue region) is performed by a pneumatic logic circuit. Eight outputs (labeled Memory bits 0 through 7) provide access to the eight stored pressure levels in the pneumatic RAM and are connected to eight fingers in two soft robotic hands.

The equivalent functional logic diagram for our pneumatic eight-bit RAM is shown in Fig 4. The pneumatic RAM is controlled by four computer-controlled solenoid valves, shown in orange in Fig 4. Three of these solenoid valves provide the values of three Address bits, which are used to select which Memory bit in the pneumatic RAM to set, and the fourth solenoid valve provides the value of the Data bit which is stored in the selected Memory bit. This is the only off-chip control hardware required to operate the pneumatic RAM; all of the remaining logic operations in Fig 4 (blue box) are performed by the pneumatic RAM chip. The pneumatic RAM chip’s logical operations include a pneumatic demultiplexer that connects the value of the Data bit to the Memory bit selected by the Address bits, and eight D-type flip-flops which maintain the value of each Memory bit between setting events. Finally, each of the eight Memory bits can be connected to a soft robotic actuator, which is operated by the pressure or vacuum stored in the Memory bit. In this manner, our pneumatic RAM uses four computer-controlled solenoid valves to control eight independent soft robotic actuators. In general, this approach can control 2^*n*−1^ independent actuators using *n* computer-controlled solenoid valves.

**Fig 5:**
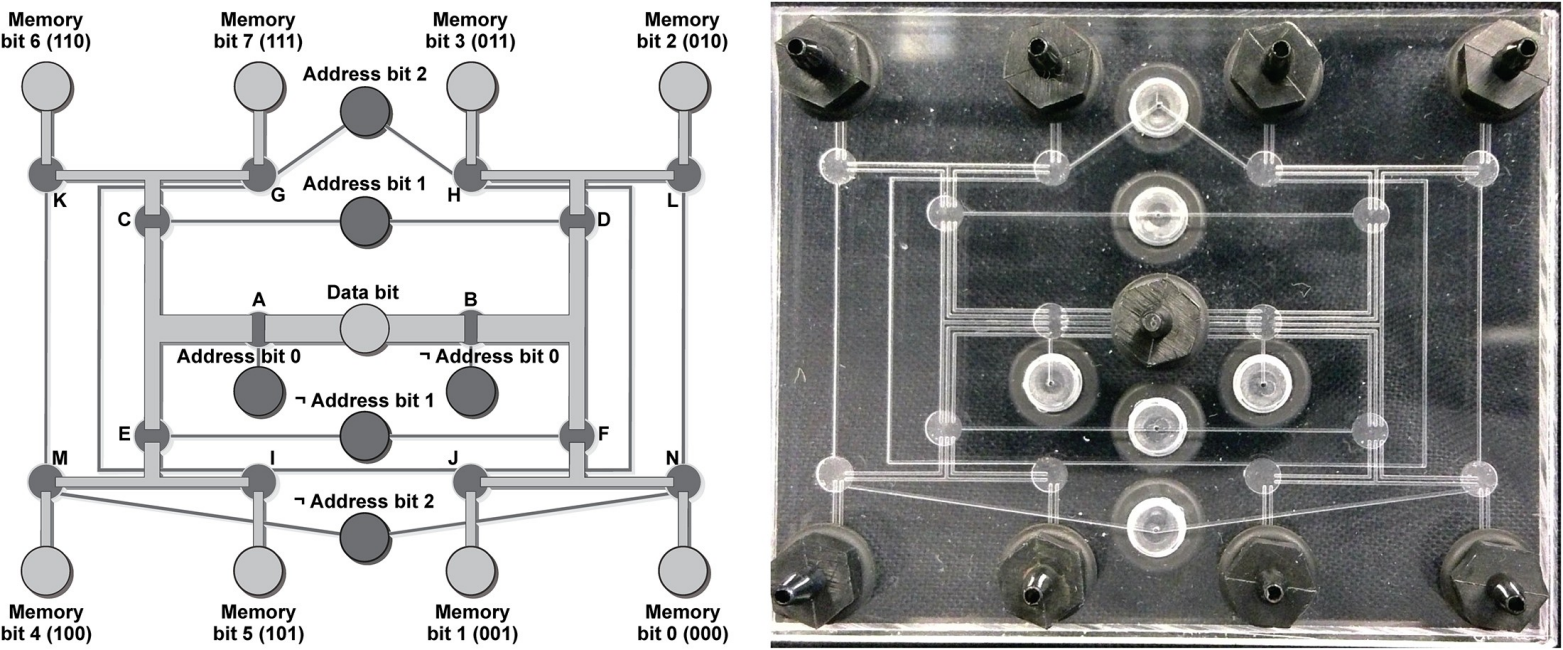
Design and photograph of a pneumatic eight-bit RAM for controlling soft robotic fingers. The 6.35 cm × 5.08 cm chip includes eight ports for connecting fingers to the Memory bits, a single Data bit connection that sets the state (contracted or extended) of each finger, three Address bit connections (and their negations, marked with “¬”) which are used to select which Memory bit (and therefore which finger) to set, and 14 pneumatic valves (labeled A through N) that execute the logical functions shown in the blue box in Fig 4.

The physical layout of the pneumatic eight-bit RAM chip is shown in Fig 5 (design file available as online *Supplementary Information*). The pneumatic RAM chip contains 14 high-flow monolithic membrane valves arranged in a pneumatic logic circuit that is functionally equivalent to the blue region in the logic diagram in Fig 4. The fact that only 14 monolithic membrane valves are needed to perform the functions of a logic diagram containing 48 logic gates is remarkable; it is a direct consequence of the normally closed nature of these valves, which enables a *single valve* to trap and “remember” a pneumatic signal and therefore function as a five-gate D-type flip-flop.

### Operation of the pneumatic eight-bit RAM

**Fig 6:**
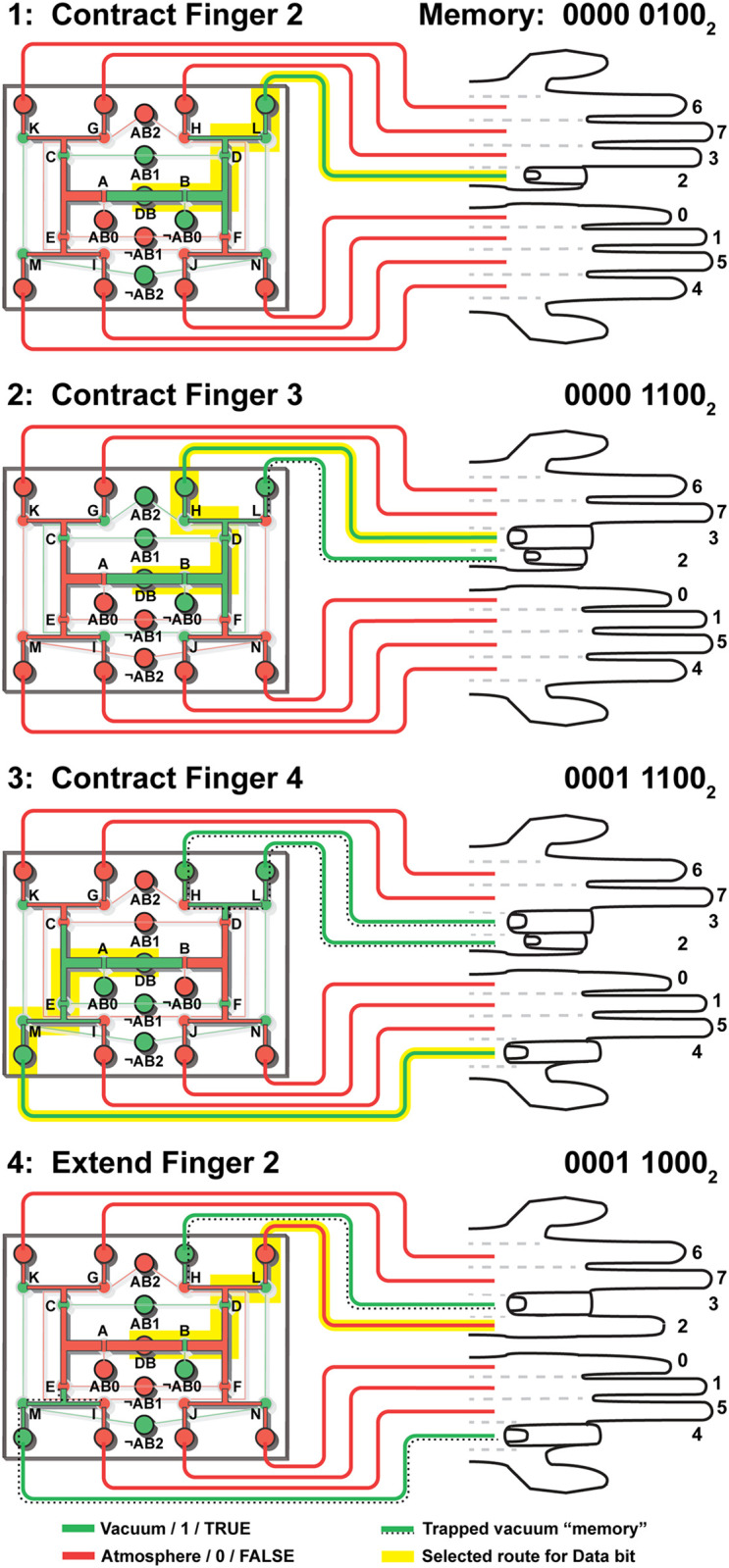
Pressure inside each channel (either green for vacuum, or red for atmospheric pressure) while using the pneumatic eight-bit RAM to contract and extend several soft robotic fingers. In each step, the vacuum or pressure applied to the Address bits (AB0, AB1, AB2, and their negations ¬AB0, ¬AB1, ¬AB2) determine the route (highlighted in yellow) followed by the Data bit (DB) signal to the selected finger. Channels containing trapped vacuum “memories” are marked using a dotted line, and the binary representation of the contents of the pneumatic RAM is shown. A detailed explanation of each step is provided in the text, and a table showing the state of each valve (A through N) during each step and a video recording of the chip during operation are available as online *Supplementary Information*.

In Fig 6 and the following paragraphs, we step through the process of using the pneumatic RAM chip to contract and extend several soft robotic finger. Additionally, a table showing the state of each valve at each step and a video recording of a pneumatic RAM chip during operation are available as online *Supplementary Information*.

The pneumatic RAM uses atmospheric-pressure air to represent a “0” or FALSE value, and vacuum to represent a “1” or TRUE value. The soft robotic fingers are extended when connected to atmospheric pressure and contracted when connected to vacuum. Initially, all of the channels inside the pneumatic RAM are at atmospheric pressure. This means that the eight stored pneumatic memory bits default to atmospheric pressure (all 0 or FALSE), and the pneumatic RAM currently stores the value 00000000_2_ (the subscript “2” indicates that this number is in base-2 or binary). The eight soft robotic fingers connected to these bits are also under atmospheric pressure and are therefore initially all extended.

#### Step 1: Contract Finger 2

In Step 1 of Fig 6 we use the pneumatic eight-bit RAM to contract Finger 2. This finger is connected to Memory bit 2, which has the address 010_2_; this means that to select Finger 2, we need to set Address bit 0 to 0 (atmosphere), Address bit 1 to 1 (vacuum), and Address bit 2 to 0 (atmosphere). The negated Address inputs (marked with a “¬” or NOT sign) automatically receive the opposites of these values, so ¬ Address bit 0 is set to 1 (vacuum), ¬ Address bit 1 is set to 0 (atmosphere), and ¬ Address bit 2 is set to 1 (vacuum). All of these Address bit values are supplied to the pneumatic RAM using three solenoid valves. Once the Address bits have been set to address Finger 2, vacuum is applied to the single Data connection in the center of the pneumatic RAM chip. This vacuum follows a path determined by the valves opened by the Address inputs, first through Valve B (which is held open by the vacuum applied to ¬ Address bit 0 according to State *VVA* in Fig 3), then through Valve D (which is held open by the vacuum applied to Address bit 1; State *VVA*), then finally through Valve L (which is held open by the vacuum applied to ¬ Address bit 2; State *VVA*). The Data vacuum then reaches Finger 2. To illustrate the logic levels inside the device, in Fig 6 (and in subsequent figures) we colored channels and tubes containing atmospheric pressure air (FALSE or 0) in red and vacuum (TRUE or 1) in green. The route followed by the vacuum from the Data bit to Memory bit 2 and Finger 2 is highlighted yellow in Fig 6 Step 1.

Finger 2 was initially filled with air at atmospheric pressure. When the pneumatic RAM routes vacuum from the Data bit to Memory Bit 2 using the process described above, this vacuum pulls air out of the finger and lowers the air pressure inside the finger; this causes the finger to contract as desired. As the air pressure inside the finger drops, it ultimately reaches a vacuum level equal to the vacuum used to control the pneumatic RAM. At this point, Valve L is no longer in State *VVA*; rather, it now is in State *VVV*. As explained in Fig 3, in State *VVV* a valve no longer has a pressure differential holding it open, so the valve closes. This transition of Valve L from open (*VVA*) to closed (*VVV*) happens automatically after the air pressure inside the finger drops to the vacuum level, and it also happens in Valves D and B, which also close automatically. In this manner, the vacuum causing Finger 2 to contract is now trapped inside the finger, separated from the rest of the pneumatic RAM chip by valves that closed automatically when the finger was done contracting. At this point, the pneumatic RAM chip now stores the value 0000 0100_2_.

Close inspection of Fig 6 reveals that in addition to the valves (B, D, and L) that are opened in Step 1 to create a path for vacuum to flow to Finger 2, four other valves also open (Valves C, K, M, and N; due to State *AVA*). However, seven other valves remain closed (Valves A, F, and H due to State *VAA* or *AAV*; and valves E, G, I, and J due to State *AAA*). These seven closed valves block the flow of air through the four open valves, so the only path for vacuum to flow through the chip is the intended path (through valves B, D, and L, to Finger 2). (As an aside, we acknowledge that our terminology of a “flowing vacuum” is nonstandard, but we nevertheless find it helpful to sometimes describe a pneumatic signal as a “vacuum flowing from A to B” rather than “air flowing from B to A”).

#### Step 2: Contract Finger 3

Next, in Step 2 of Fig 6 we wish to contract Finger 3 while keeping Finger 2 contracted (in other words, we need to transition the value stored by the pneumatic RAM from 0000 0100_2_ to 0000 1100_2_). To accomplish this, we apply the address of Memory bit 3 (011_2_) to the Address bits: Address bit 0 = 1 (vacuum), Address bit 1 = 1 (vacuum), and Address bit 2 = 0 (atmosphere), along with their opposites to the negated Address bits: ¬ Address bit 0 = 0 (atmosphere), ¬ Address bit 1 = 0 (atmosphere), and ¬ Address bit 2 = 1 (vacuum). This opens a path (colored yellow in Fig 6 Step 2) for vacuum to flow from the Data connection, through Valves B, D, and H (all held open in state *VVA*), to Finger 3 which then contracts. As in Fig 2 above, when the contents of Finger 3 reach vacuum, Valves B, D, and H all close due to the automatic transition from State *VVA* to State *VVV*.

What keeps the original Finger 2 contracted (under vacuum) while the pneumatic RAM chip is setting the new Finger 3? As shown in Step 2, Valve L (which leads to Finger 2) is in State *VAV*, with vacuum from the pneumatic RAM chip’s Data bit applied to the valve’s Input, atmospheric pressure applied to the valve’s Control, and the vacuum trapped inside the finger present at the valve’s Output. State *VAV* dictates that this valve will remain closed, so the vacuum remains trapped inside Finger 2 and the channels and tubing leading up to it (this trapped vacuum “memory” is marked with a dotted line in Fig 6 Step 2). This trapped vacuum keeps Finger 2 contracted, even while the pneumatic RAM is contracting Finger 3. At the conclusion of this step, the the pneumatic RAM now stores the value 0000 1100_2_.

#### Step 3: Contract Finger 4

Next, in Step 3 of Fig 6, we wish to contract Finger 4 while keeping Fingers 2 and 3 contracted. This corresponds to transitioning the value stored in the pneumatic RAM from 0000 1100_2_ to 0001 1100_2_. As before, we apply the address of Memory bit 4 (100_2_) to the pneumatic RAM’s Address bits and the negated Address bits. This opens a path (colored yellow) for vacuum to flow from the Input connection, through valves A, E, and M (held open in state *VVA*), to Finger 4 which then contracts. Again as before, Valves A, E, and M then automatically close (transitioning from *VVA* to *VVV*) when the contents of Finger 4 reach vacuum.

At this point, Fingers 2 and 3 both need to remain contracted while the pneumatic RAM chip is contracting Finger 4. Inspection of Step 3 in Fig 6 shows how this is possible. Valve L, which was previously closed and used to trap the vacuum inside Finger 2, is actually *open* in Step 3 because it shares a Control line with Valve M (which had to be opened to send vacuum to Finger 4). However, Finger 2 remains contracted because the valves upstream of Valve L in the pneumatic RAM, Valves H and D, are both closed. Thus, while a small amount air from the channels between Valves L, H, and D does flow toward the vacuum trapped in Finger 2 in Step 3, the volume of this air is negligible compared to the volume of vacuum trapped inside Finger 2, so the vacuum inside Finger 2 remains virtually unchanged and the finger remains contracted. Similarly, the vacuum inside Finger 3 remains trapped by Valve H being closed in Step 2. At the end of this step, the pneumatic RAM’s contents are 0001 1100_2_.

#### Step 4: Re-extend Finger 2

Finally, in Step 4 of Fig 6, we wish to re-extend Finger 2 while keeping Fingers 3 and 4 contracted (that is, transition the pneumatic RAM’s contents from 0001 1100_2_ to 0001 1000_2_). To extend Finger 2, the pneumatic RAM chip needs to route atmospheric pressure to the finger; the resulting air flow into the finger destroys the trapped vacuum and resets the finger to its resting (extended) state. To accomplish this, we again apply the address of Memory bit 2 to the pneumatic RAM’s Address bits and negated Address bits. This opens a path (colored yellow) for atmospheric pressure to flow from the Data bit connection, through valves B, D, and L (now held open in State *AVV*), to Finger 2, thereby extending the finger.

Once more, inspection of Step 4 reveals why Fingers 3 and 4 remain contracted while the pneumatic RAM extends Finger 2. Finger 3’s trapped vacuum is sealed by Valve H, which is closed in Step 4. Finger 4’s trapped vacuum is no longer sealed by Valve M, which was opened in Step 4 as a consequence of opening Valve L which shares a Control line with Valve H; however, the neighboring Valves I and E *are* closed in Step 4, and those valves seal the trapped vacuum inside Finger 3 during Step 4. The pneumatic RAM now stores the value 0001 1000_2_.

In this manner, the pneumatic RAM can set soft robotic fingers to any desired combination of contracted or extended states, and the fingers will “remember” their state until they are set to a different state.

### Testing the pneumatic RAM

After fabricating our pneumatic RAM, we made eight 3D-printed soft robotic fingers (details in *Materials and Methods* below) to use in testing the pneumatic RAM. These flexible elastomer fingers are normally extended when their hollow interiors are under atmospheric pressure. When a vacuum is applied to a finger, the finger contracts and curls into a C-shape. Restoring atmospheric pressure to the finger causes it to extend again. We used these fingers in a series of tests to confirm that the pneumatic RAM operates as intended when controlling a soft robot.

**Fig 7:**
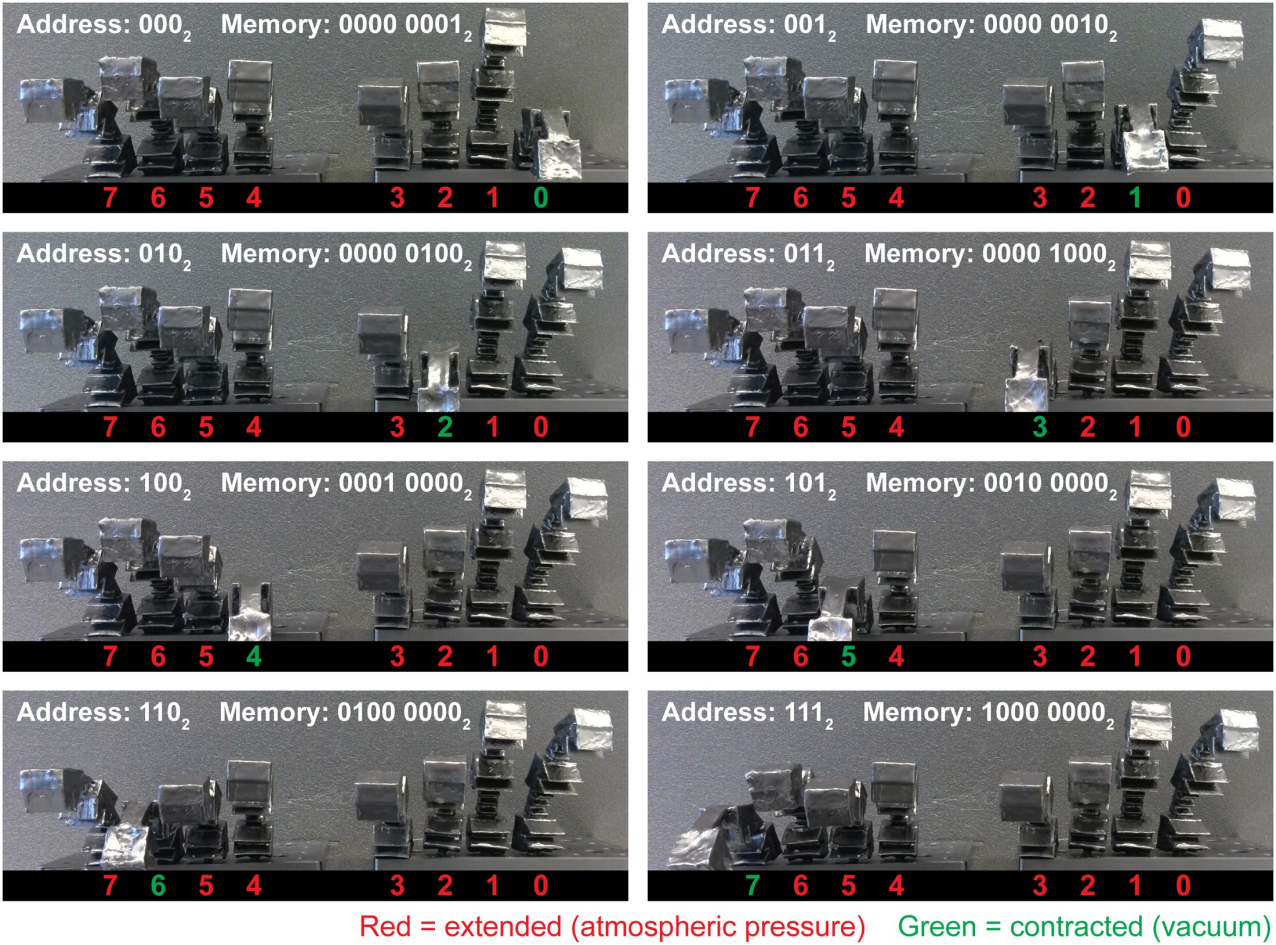
Video frames from using the pneumatic eight-bit RAM to contract eight soft robotic fingers one-at-a-time. The pneumatic RAM cycles through all eight addresses from 000_2_ to 111_2_ to set the pneumatic RAM’s memory to the values shown (from 0000 0001_2_ to contract just Finger 0, to 1000 0000_2_ to contract just Finger 7).

In the first phase of this testing, we used the pneumatic RAM to contract one finger at a time while holding the other fingers extended. Frames from a video recording of the experiment are shown in Fig 7. As the pneumatic RAM steps through all eight possible values for the three Address bits (from 000_2_ for Memory bit 0, to 111_2_ for Memory bit 7), the finger connected to each Memory bit contracts, thereby confirming that the Data bit vacuum was successfully routed to each finger in turn.

After demonstrating that the pneumatic RAM chip can control each finger independently, we then tested whether the pneumatic RAM can set and maintain all eight fingers in any desired pattern of contracted and extended fingers. This is a considerably more difficult task for the pneumatic RAM chip: there are 2^8^ or 256 different patterns of contracted or extended fingers, ranging from all eight extended (0000 0000_2_) to all eight contracted (1111 1111_2_) and every combination in between, and each finger must “remember” its state (contracted or extended) while other fingers are being set.

**Fig 8:**
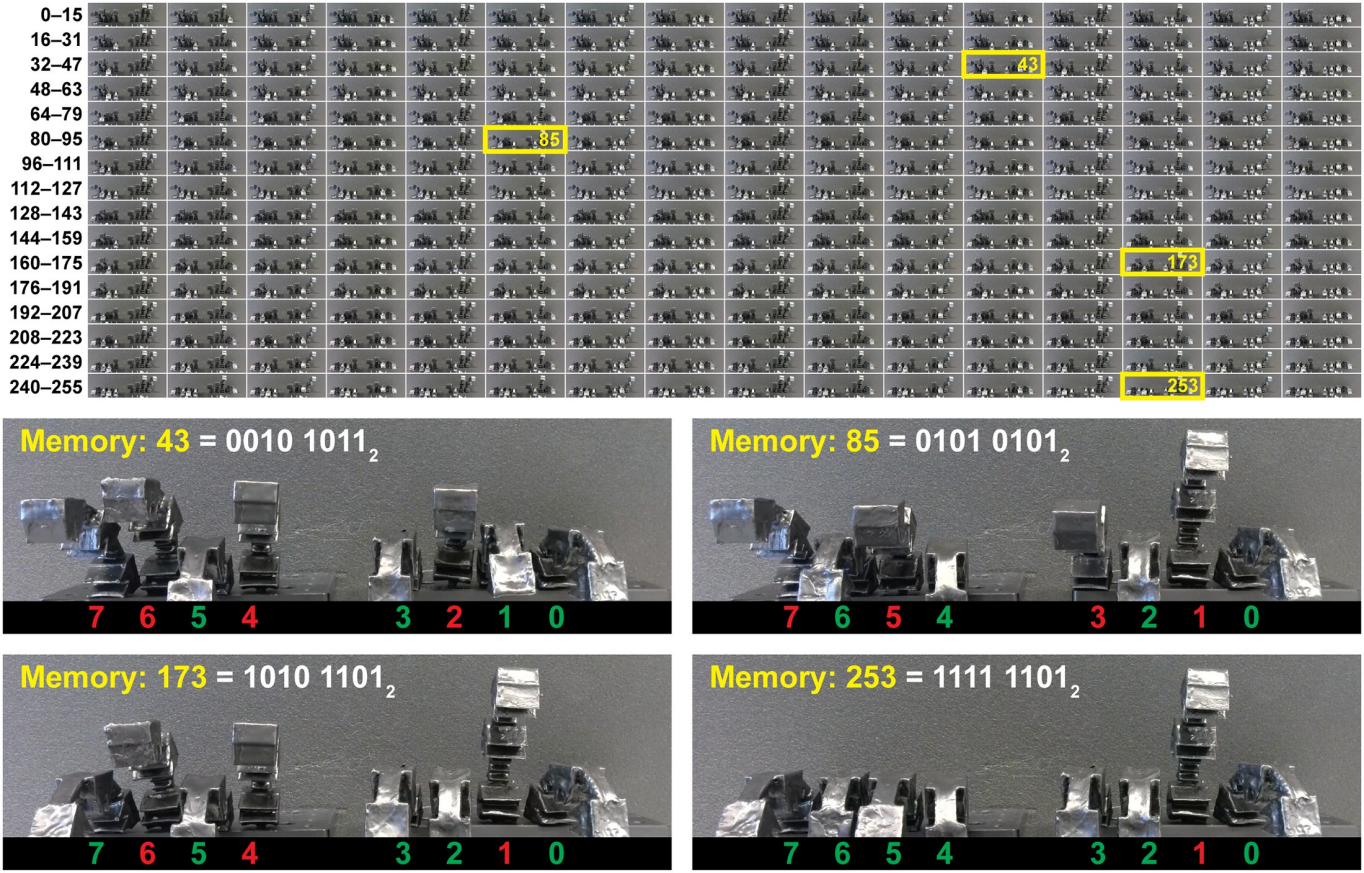
Video frames from using the pneumatic RAM to set eight soft robotic fingers to all 256 different possible patterns, ranging from all extended (corresponding to a value of 0000 0000_2_ or 0 stored in the pneumatic RAM) to all contracted (a value of 1111 1111_2_ or 255 stored in the pneumatic RAM), with closeups of the fingers while the pneumatic RAM is storing the values 43, 85, 173, and 253. Closeups of all 256 frames are available as *Supplementary Information*.

Fig 8 shows frames from a video recording of the pneumatic RAM setting all eight fingers to all 256 possible patterns. The video starts with all eight fingers extended, corresponding to a value of 0000 0000_2_ (or 0 in decimal) stored in the pneumatic RAM. Next, the pneumatic RAM uses the address of Memory bit 0 (000_2_) to route a vacuum that contracts Finger 0. This transitions the value stored by the pneumatic RAM to 0000 0001_2_ (or 1 in decimal). In the next step, the pneumatic RAM uses the address of Memory bit 1 (001_2_) to route a vacuum that contracts Finger 1, then uses the address of Memory bit 0 again (000_2_) to contract Finger 0; this transitions the pneumatic RAM contents to 0000 0010_2_ (or 2 in decimal). This process is continued in a binary counting pattern—0000 0011_2_ (or 3 in decimal), 0000 0100_2_ (or 4 in decimal), 0000 0101_2_ (or 5 in decimal), and so on—through all 256 possible states of the pneumatic RAM contents, all the way to 1111 1111_2_ (255 in decimal). Photographs of the fingers in all 256 different memory states are shown in Fig 8, along with closeups corresponding to the pneumatic RAM storing the values 43 (0010 1001_2_), 85 (0101 0101_2_), 173 (1010 1101_2_), and 253 (1111 1101_2_). In addition, closeups of the fingers during all 256 different states of the memory contents are available as *Supplementary Information*. No errors were observed during the experiment. This confirms that the pneumatic RAM chip can remember 256 different values and use these values to set and maintain eight soft actuator fingers according to any of 256 different actuation patterns.

### Characterizing pneumatic “memory”

As noted above, the pneumatic RAM can control 2^*n*−1^ independent soft robotic actuators using *n* computer-controlled solenoid valves. This exponential relationship means that an extremely large number of actuators can be controlled by a modest amount of control hardware. But since the pneumatic RAM can only update the state of one actuator at a time, as the number of actuators grows, the amount of time between updates for a given actuator also increases. Limited update frequency is not an issue for an actuator being held at atmospheric pressure (FALSE or 0) because atmospheric pressure is the normal at-rest state in our pneumatic RAM chip; it can maintain actuators at atmospheric pressure indefinitely. However, update frequency *is* important for an actuator being held under vacuum (TRUE or 1) because small unintended air flows (due to *e.g*. small volumes of atmospheric-pressure air routed to the actuator during routine pneumatic RAM operation as described above) could deplete the trapped vacuum over time. Consequently, the trapped vacuum that “remembers” the state of an actuator needs to last for as long as possible, at least long enough to maintain the state of an actuator until the pneumatic RAM refreshes the vacuum during its next cycle of setting the actuators.

**Fig 9:**
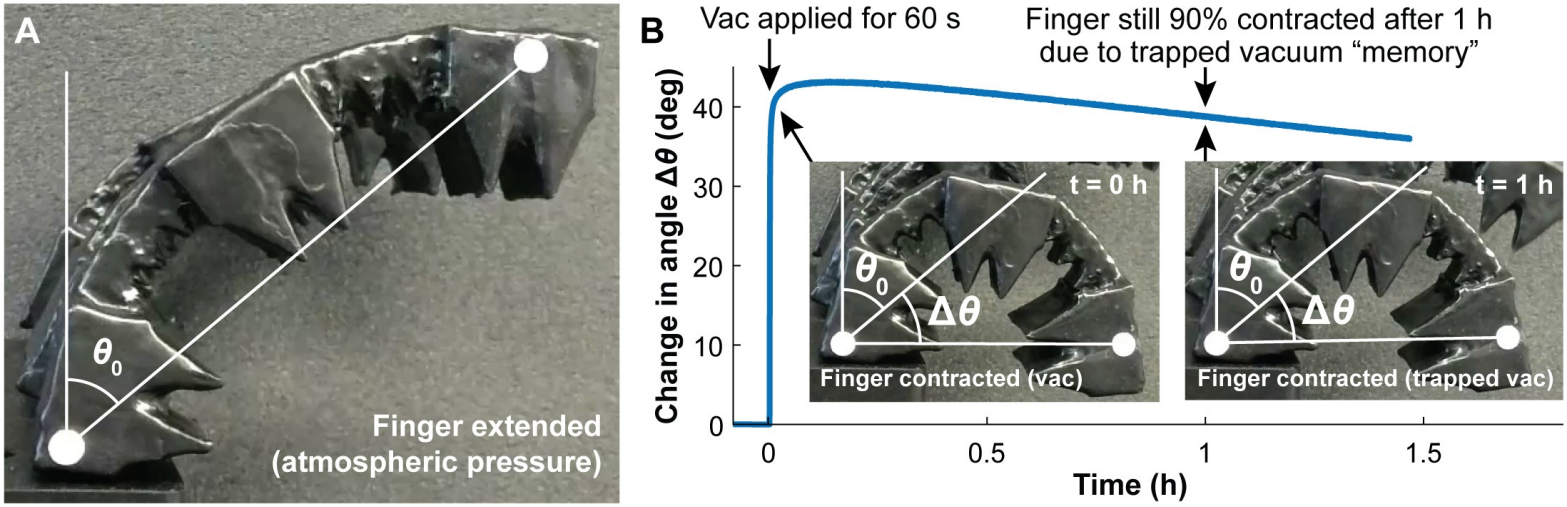
Measuring the amount of time that the pneumatic RAM can “remember” the value of a Memory bit (and how long a soft robotic finger connected to the Memory bit can maintain its actuation state). **(A)** At atmospheric pressure, the finger has a natural curve with an angle *θ*_0_. **(B)** At time = 0, the pneumatic RAM applies vacuum from the Data input to the Memory bit and the finger for an excessively-long 60 s, which causes the finger to contract an additional Δ*θ* degrees. After 60 s the pneumatic RAM disconnects the Data vacuum from the Memory bit, but the finger remains contracted due to the vacuum trapped in the Memory bit and inside the finger. For the next hour, the pneumatic RAM cycled through three other fingers, contracting and extending one every ten seconds. Even though the pneumatic RAM chip’s operation introduces small amounts of atmospheric-pressure air to the first finger, the finger nonetheless remained 90% contracted 1 h after the applied vacuum was removed. Therefore, a trapped vacuum at a pneumatic RAM Memory bit can hold a finger contracted for at least an hour before needing to be “refreshed” by the pneumatic RAM. A closeup view of the first few seconds of this plot is available as online *Supplementary Information*.

To determine how long a latched vacuum can hold a soft robotic finger in the contracted state, we first developed a method for measuring the amount of finger contraction. Our 3D-printed soft robotic fingers normally have a slight curvature when extended; this curvature gives rise to the angle *θ*_0_ shown in Fig 9A. When a vacuum is applied to the finger, the finger contracts, increasing its curvature by an additional angle Δ*θ* as shown in the inset images in Fig 9B. To measure and monitor the degree of contraction over time in a finger containing a latched vacuum, we wrote a MATLAB script (available as online *Supplementary Information*) that extracts *θ*_0_ and Δ*θ* from a video recording of the side view of the finger.

We then wrote a program for the pneumatic RAM that subjects a finger to a “stress test” intended to determine how long one finger can remain contracted by a trapped vacuum from a Memory bit while the pneumatic RAM is actively controlling other fingers. This program initially routes a vacuum to a finger for 60 seconds, during which time the finger contracts and the vacuum is sealed inside the finger when the pneumatic RAM chip’s valves automatically close, as described above. Applying vacuum for an excessively-long 60 seconds ensures that the finger is fully contracted. Then, the program uses the pneumatic RAM to continuously contract and extend the other three fingers connected to the IC. This is a “stress test” because, while the pneumatic RAM is applying vacuum and atmospheric pressure to the other fingers, small amounts of atmospheric-pressure air from inside the pneumatic RAM chip’s channels are leaked to the trapped vacuum in the first finger (this is a normal side-effect of pneumatic RAM operation, as explained in the description of Fig 6 above). If enough atmospheric-pressure air enters the first finger, it could degrade or even eliminate the trapped vacuum in the Memory bit that is holding the finger contracted, effectively causing the pneumatic RAM to “forget.”

A typical result obtained from subjecting a soft robotic finger to this pneumatic memory “stress test” is shown in Fig 9B. The slow decrease in the measured contraction angle Δ*θ* confirms that some atmospheric-pressure air is entering the trapped vacuum inside the finger while the pneumatic RAM chip is operating other fingers. However, the finger is still 90% contracted one hour after the pneumatic RAM routed the original vacuum to the finger. This was the median result of the three soft robotic fingers we used in this test; in the other experiments (available in online *Supplementary Information*) the pneumatic RAM kept the fingers at least 90% contracted for 22 minutes and 1.2 hours. These results show that the Memory bits on the pneumatic RAM can maintain the state of a soft robotic finger for extended periods of time before needing to be “refreshed.”

A closeup of the first few seconds of the plot in Fig 9B (available as online *Supplementary Information*) shows that the finger is 70% contracted after just 4 seconds, 80% in 7 seconds, and 90% contracted in 18 seconds. For many applications, 70% contraction of an actuator may be adequate, so each actuator could be set in 4 seconds and remember its state for at least an hour. In this manner, a pneumatic RAM could control 900 independent actuators, updating the state of each actuator every hour. To control this massive number of actuators, the pneumatic RAM would require only log_2_ 900 + 1 = 11 solenoid valves (ten to set the ten Address bits and their negations, and one to set the Data bit); this represents a *99% reduction* in the amount of control hardware that would normally be required to operate 900 independent soft robotic actuators. For applications that require more frequent updates of actuator state, one can halve the number of independent actuators and double the update frequency. So, a pneumatic RAM could set 450 actuators to any desired actuation pattern every 30 minutes, or set eight actuators to any pattern every minute, and so on.

Note that the projections above assume that the pneumatic RAM sets every actuator during each operation cycle. This is not strictly required, and for many applications it would be advantageous to set some actuators more frequently than others. In this manner, a pneumatic RAM could support both rapidly operating actuators (updated every few seconds) and less-frequently operated actuators (updated only when needed) on the same robot. Additionally, the subset of actuators that require the most rapid operation can always be connected directly to solenoid valves as is typically done, with the remaining actuators controlled via a pneumatic RAM.

### Pneumatic digital-to-analog conversion

The analysis above treats our soft robotic fingers as binary (either contracted or extended). However, it is noteworthy that the pneumatic RAM can also maintain these fingers at any intermediate position. This can be accomplished by using the pneumatic RAM to set the pressures at the Memory bits to intermediate values between full atmospheric pressure (corresponding to full extension) and full vacuum (corresponding to full contraction). Specifically, by changing the amount of time that the pneumatic RAM applies full vacuum or full atmospheric pressure from the Data input to a Memory bit, the pneumatic RAM can set an arbitrary pressure in Memory bit and therefore set the connected actuator to any desired angle. For example, having the pneumatic RAM deliver vacuum to a Memory bit for about 2.5 s duration sets the associated finger about halfway between the contracted and extended states (see *Supplementary Information*). In this manner, the pneumatic RAM can function like a multi-channel digital-to-analog converter and data buffer [39], setting multiple soft robotic actuators to any desired position (analog) using different-duration pulses of a constant vacuum or atmospheric pressure (digital), and maintaining the actuators in those positions for an extended period of time. The accuracy and precision of this pneumatic digital-to-analog conversion would depend upon the actuators having predictable and consistent behavior, so this method may not be suitable for precision control, but it nonetheless demonstrates that the pneumatic RAM is not strictly limited to just binary (either contracted or extended) control of actuators.

### Simultaneous actuation of multiple fingers

In the tests described above, our pneumatic RAM was limited to changing the state of one soft robotic finger at a time. Since the fingers can “remember” their state thanks to trapped pressures in the pneumatic RAM’s Memory bits, this one-at-a-time operation does not limit the number of finger actuation patterns that are possible when multiple fingers are controlled by the same pneumatic RAM. However, there are many situations where it would be desirable to change two or more fingers *simultaneously*. For example, a biomimetic soft robotic hand might contract all five of its fingers simultaneously to grasp an object, then extend all of its fingers simultaneously to release it. If two or more actuators are *always* to be operated in lock-step, then they could be connected to the same Memory bit on the pneumatic RAM; this would guarantee true simultaneous actuation at the expense of individual control. However, for applications that require both individual *and* simultaneous control of actuators, a different solution is needed.

To solve this problem, we again turned to techniques originally developed for electronic circuits. In this case, the principle of *time-division multiplexing* (TDM) allows electronic circuits to send multiple messages simultaneously through a single communication channel [39]. In TDM, a switch continuously and rapidly alternates between the original signals, sending only part of each message at a time. When these parts are reconstructed on the other end of a communication channel, the full messages are recreated.

Inspired by electronic TDM, we hypothesized that by operating the pneumatic RAM at high speed, we could use it to send brief pulses of atmospheric pressure or vacuum to each Memory bit and each connected finger. The duration of each pulse would be short, too short for a single pulse to fully contract or extend a finger. However, as multiple pulses are delivered to each finger, their effects would accumulate and ultimately contract or extend the fingers. By cycling through the fingers rapidly in a manner akin to electronic TDM, the pneumatic RAM could contract or extend several fingers essentially simultaneously.

To test this idea, we needed a task for a soft robot that requires multiple fingers to be actuated simultaneously. We decided that playing a piano keyboard was a suitably complex task because piano playing requires pressing not only individual keys but also multiple keys at once to play chords. The sound produced when a key is pressed indicates that the attempt to press the key has been successful.

**Fig 10:**
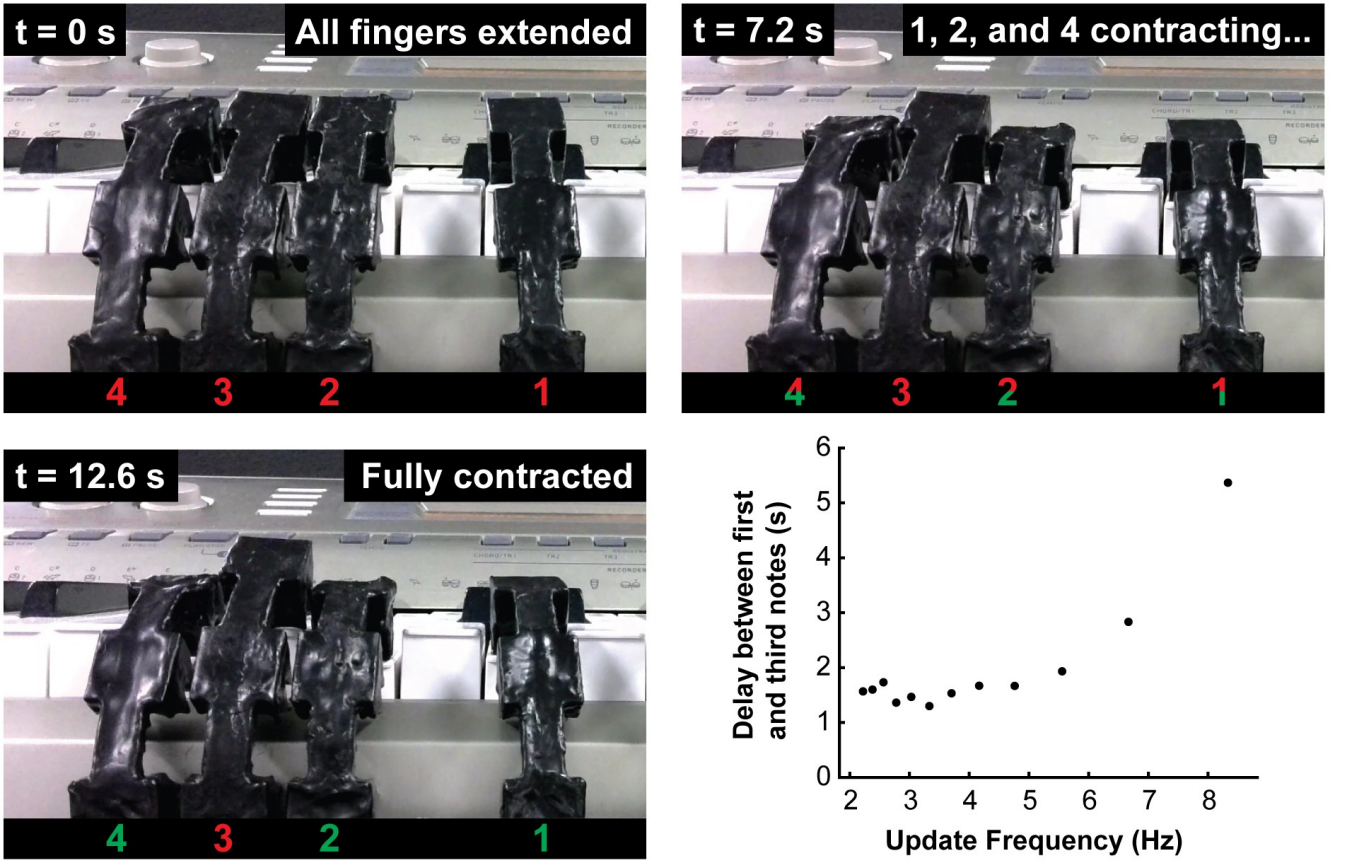
Video frames from using the pneumatic RAM and principles of time-division multiplexing (TDM) to control soft robotic fingers simultaneously playing the notes G, B, and D (a G-major chord) on an electric piano keyboard. The plot shows the observed delay between the start of the first note of the chord and the last note of the chord, as a function of the cycle speed of the pneumatic RAM. At cycle frequencies above 5.56 Hz the pneumatic RAM’s performance degrades and the chord’s notes do not sound simultaneously, but below 5.56 Hz the notes sound within 1 to 2 seconds of each other.

As shown in Fig 10, we mounted a set of four soft actuator fingers in front of an electric piano keyboard and wrote a program that makes the pneumatic RAM constantly deliver brief pulses of vacuum from the Data input to Memory bits 1, 2, and 4, which in turn were connected to three of the four fingers (the fourth finger received no vacuum pulses). The amount of time the pneumatic RAM connected each Memory bit to vacuum was varied from 150 ms down to only 40 ms. With three fingers updated in each cycle of the pneumatic RAM, these times correspond to cycle times ranging from 450 ms to 120 ms, meaning that each finger receives from 2.2 to 8.3 vacuum pulses per second.

Fig 10 shows frames from a video recording of a typical experiment using our soft robotic fingers to play the notes G, B, and D simultaneously (a G-major chord) under the control of the pneumatic RAM using TDM. In this experiment, the pneumatic RAM connected each finger to vacuum for 40 ms, cycling through all three fingers every 120 ms, or an update frequency of 8.33 Hz. At time *t* = 0, the pneumatic RAM has not yet begun operating and all four fingers are extended. After 7.2 s of operating the pneumatic RAM under TDM, Fingers 1, 2, and 4 are visibly contracting. By *t* = 12.6 s, all three fingers are fully contracted and the G-major chord is audible.

Ideally, in these experiments, all three notes in the G-major chord should sound simultaneously. In practice, at the fastest update frequency of 8.33 Hz, we observed up to 5 s of delay between the start of the first note in the chord and the start of the last note (Fig 10). This suggests that at the fastest update frequencies, the pneumatic RAM is simply running too fast for reliable operation. However, at update frequencies of 5.56 Hz and lower, the delay between the first and third notes in the chord drops to approximately 1 to 2 seconds. While still not simultaneous, the fingers are nonetheless contracting together in a reasonable amount of time, and this timing should be adequate for activities like grasping and releasing an object.

### Playing a song on the piano

**Fig 11:**
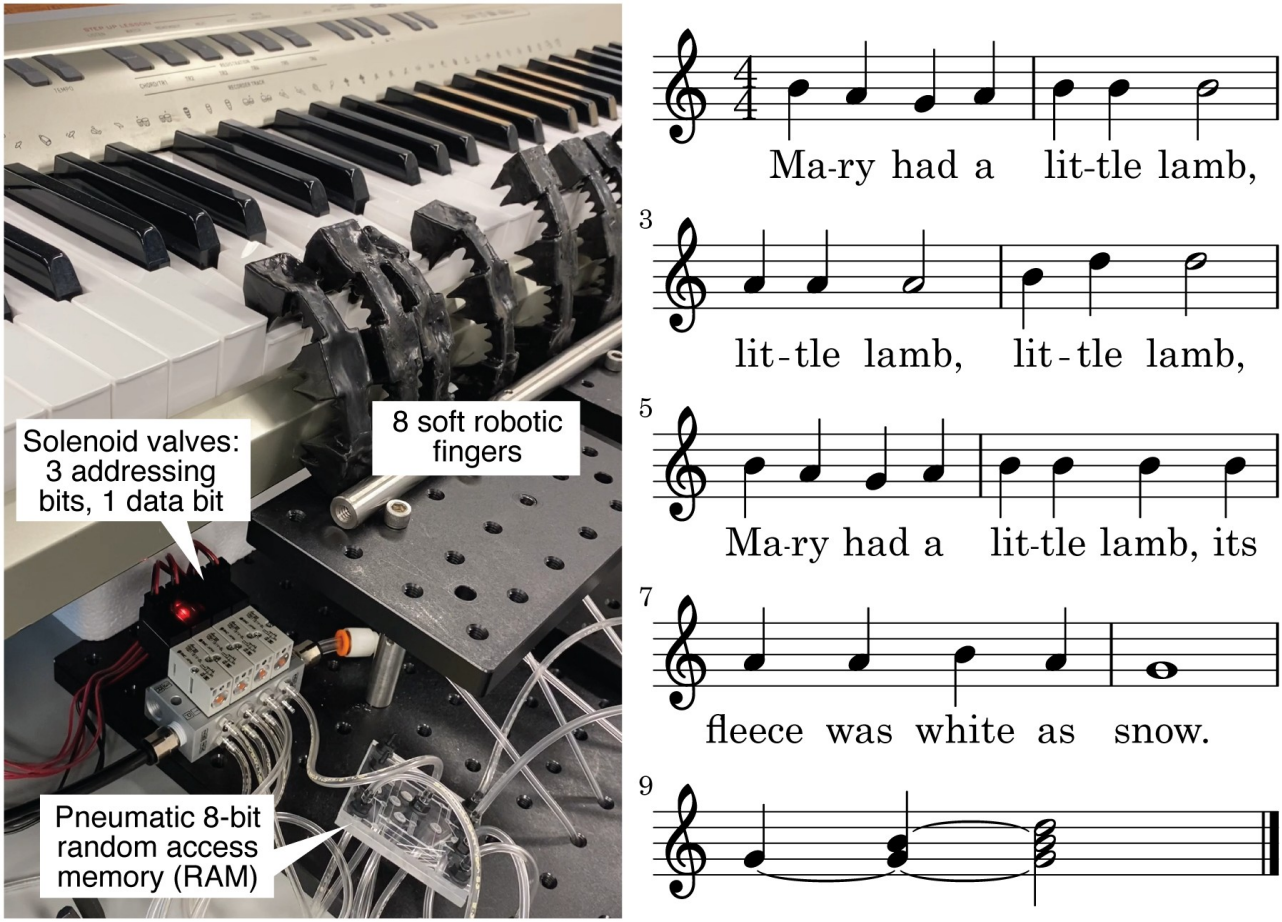
Video frame from using an eight-bit pneumatic RAM to control soft robotic fingers playing the music score shown on the right. The music consists of “Mary Had a Little Lamb” (measures 1 through 8; demonstrating playing one note at a time with varying durations) followed by an arpeggiated G-major chord (measure 9; demonstrating playing and holding multiple notes simultaneously). Source video available as online *Supplementary Information*.

Finally, to demonstrate that the pneumatic RAM can control fingers both individually and simultaneously in a complex actuation pattern, we programmed the chip to play a song on the electric piano keyboard, in a manner akin to past demonstrations of piano-playing soft robots [40]. We wrote the arrangement of “Mary Had a Little Lamb” shown in the musical score in Fig 11 and programmed the pneumatic RAM to play the song. A video recording of the performance (available as online *Supplementary Information*) shows that the soft robotic fingers played the song successfully and no errors were observed.

## Discussion

In this work, we introduced a pneumatic nonvolatile random-access memory (RAM) and showed that this pneumatic logic circuit can dramatically reduce the amount of peripheral hardware required to control a soft robot. To conclude, we examine the capabilities, practical limitations, and future directions for this technology.

Based on our experimental findings, we noted earlier that a 10-bit pneumatic RAM could set 900 independent actuators to any desired actuation pattern. Would a pneumatic RAM chip this complex be feasible? To answer this, we can estimate some design parameters for this chip. In general, an *n*-bit pneumatic RAM capable of controlling 2^n^ independent soft actuators will contain 2^*n*+1^ − 2 on-chip valves, so a 10-bit pneumatic RAM would contain 2046 on-chip monolithic membrane valves. With a surface area of about 7 mm^2^ for each of our current valves, a 10-bit pneumatic RAM would contain about 140 cm^2^ of valves; this is about the same surface area as a modern smartphone screen. While probably too large for some applications, a smartphone-sized pneumatic RAM would nonetheless be suitable for controlling many medium- and large-scale soft robots. And by using smaller monolithic membrane valves [36] or stacking several smaller pneumatic RAM chips, the overall footprint of the pneumatic RAM could be significantly reduced. Most importantly, this pneumatic RAM would require only 11 solenoid valves to operate it, a *99% reduction* in the amount of bulky, expensive, and power-consuming electromechanical hardware required to control a soft robot with 900 independent actuators. Since pneumatic soft robots usually require multiple solenoid valves (one per control line), a technology that exponentially reduces the number of solenoid valves required to operate a soft robot (while still maintaining complete individual control of each actuator) will result in significant reductions in the overall system cost, size, and power consumption of the soft robot.

For some applications, dramatically reducing the amount of electromechanical control hardware may not be enough—completely *eliminating* it would be the ultimate goal. For example, soft robotic wearable devices can help infants with motor impairments lead normal lives [6], but the computers, microcontrollers, solenoid valves, batteries, and other hardware used to control these devices are difficult to safely use in close proximity to small children. Could pneumatic logic chips replace all this hardware? Replacing traditional computers and microcontrollers would require pneumatic logic circuits that can run complex multi-step programs, and these circuits have already been demonstrated at the microfluidic scale [27, 29–36]. In principle, by replacing the monolithic membrane valves in these pneumatic computers with the high-flow valves we introduced in Fig 2C, one could make programmable pneumatic logic chips that control soft robots without the need for any electronic computing hardware. To power this pneumatic computer, we would still need a source of vacuum. This could be provided by a battery-powered pump, or for some applications a small evacuated tank could provide all of the power needed to power and control a soft robot for a period of time.

In its current form, the pneumatic RAM chip stores vacuums (pressures lower than atmospheric pressure). This means that the pneumatic RAM is limited to controlling vacuum-operated actuators like the soft robotic fingers used here. Vacuum-operated actuators are naturally limited to one atmosphere of pressure differential, and this makes them potentially “weaker” than pressure-operated actuators (which can in principle be powered by any desired pressure differential). For many applications that value the inherent safety and gentleness of soft robots, this limitation may actually be desirable: with no positive pressure anywhere in the system, there is no risk of accidental overpressurization and violent failure of the robot or its control system. However, to support the full range of pneumatic actuators, a pneumatic RAM chip that can also store *pressures* would be necessary. Monolithic membrane valve-based pneumatic logic circuits capable of storing both vacuums *and* pressures in microfluidic applications have been demonstrated previously [28]; by implementing those circuits using the high-flow monolithic membrane valves described here, a pneumatic RAM capable of controlling *any* pneumatic actuator could be possible.

Finally, pneumatic RAM chips are not limited to controlling soft robots. Many traditional “hard” robots are powered by pistons and other pneumatic actuators, as is other equipment in manufacturing, construction, agriculture, mining, and other industries. Pneumatic RAM could reduce or eliminate electromechanical control hardware in these industries as well. In addition to providing cost savings, pneumatic RAM would be particularly useful in hazardous environments like coal mines, grain silos, and chemical plants, where electronic hardware could spark and cause fires or explosions.

## Materials and methods

### Designing and fabricating pneumatic RAM chips

The pneumatic RAM chip in Fig 5 was designed in Adobe Illustrator (file available as online *Supplementary Information*), exported as a PDF file, and engraved into two pieces of poly(methyl methacrylate) (PMMA or acrylic) using a desktop CNC milling machine (Bantam Tools; Peekskill, New York). Each acrylic piece was 6.35 cm wide, 5.08 cm deep, and 3 mm thick. The channels milled into each layer were 280 *μ*m wide and 254 *μ*m deep. In the design in Fig 5, the channels connecting the central “Data bit” to the eight “Memory bit” connections (and thus to the soft robotic actuators) all have two, three, or five parallel channels to accommodate greater air flow through these lines and therefore faster operation of the soft robot’s actuators. We varied the number of parallel channels from two to five as an experiment, but in principle a chip designer can use as many parallel channels as they wish, with more parallel channels providing higher throughput of air and faster operation of the connected soft robot. Multiple parallel channels are not necessary leading to the valves’ Control channels because these channels carry only small volumes of air flowing to and from the valves’ Control chambers (not the larger volumes of air flowing to and from the soft robot’s actuators). Each high-flow monolithic membrane valve inside the chip had a circular Control chamber with a diameter of 3 mm and a depth of 254 *μ*m. The ports for the single Data bit input, six Address bit inputs, and eight Memory bit outputs had a diameter of 4.0 mm before being tapped with 10–32 threads and fitted with tubing connectors.

After engraving their channel patterns, the two PMMA pieces were bonded together with a featureless sheet of polydimethylsiloxane (PDMS) silicone rubber to form the completed pneumatic RAM. The 254 *μ*m thick PDMS sheet (HT-6240; Rodgers Corporation/Bisco Silicones, Carol Stream, IL) was treated for 45 s per side using a handheld corona treater (BD-20AC; Electro-Technic Products, Chicago, IL) before bonding; this treatment strengthens the bond between the PDMS sheet and the PMMA pieces [41]. The engraved PMMA pieces were cleaned with 99.9% isopropanol, dried, then submerged in a 1% (volume/volume) solution of 3-aminopropyltriethoxysilane (Sigma-Aldrich, St. Louis, MO) in purified water for 20 minutes; this treatment also strengthens the PDMS-PMMA bond [42]. After drying, the bonding surfaces of the PMMA pieces were corona treated for 45 seconds before sandwiching the PDMS sheet between the two PMMA pieces. The resulting pneumatic RAM chip was gently clamped and left overnight for the bond to strengthen before removing the clamp and using the chip.

### Designing and fabricating soft robotic fingers

Soft robotic fingers were designed in SolidWorks (file available as online *Supplementary Information*), exported as an STL file, prepared for 3D printing using Ultimaker Cura software (0.1 mm layer height and 100% infill density), and fabricated using a 3D printer (Ender 3; Creality 3D Technology Co., Shenzhen, China). The filament used by the printer was thermoplastic polyurethane or TPU (NinjaFlex; Fenner Inc., Manheim, PA), a flexible filament with a Shore hardness of 85A. The 3D printer used an extruder temperature of 230°C and an unheated (room temperature) print bed. After printing, small leaks in the soft robotic fingers were sealed by dipping the fingers in a rubber coating (Plasti Dip International, Blaine, MN) diluted to 50% in toluene, and then leaving the fingers to dry for at least four hours before use.

### Controlling the pneumatic RAM chip

As shown in Figs 4–6, the eight-bit pneumatic RAM is controlled by four pneumatic inputs: one Data bit input that receives vacuum or atmospheric pressure for routing to the selected Memory bit, and three Address bit inputs that determine which of the eight Memory bits (and associated soft robotic fingers) receives vacuum or atmospheric pressure. These inputs are provided by four “2 position, 4 way, 4 ported” solenoid valves (VQD1151–5L; SMC Corporation of America; Noblesville, Indiana) connected to a manifold (VV4QD15–04M5; SMC). Each valve has two inlets, one connected to house vacuum (–68 kPa) and one left open to the atmosphere (this is the source of atmospheric pressure in our pneumatic logic circuits). Each valve also has two outlets, one connected to an Address input on the pneumatic RAM, and the other connected to the corresponding negated Address (or ¬ Address) input, as shown in Fig 5. At rest (not energized), each solenoid valve applies atmospheric pressure to the pneumatic RAM’s Address input and vacuum to the ¬ Address input. When energized, the solenoid valve swaps these outputs, connecting vacuum to the pneumatic RAM’s Address input and atmospheric pressure to the ¬ Address input. In this manner, the four solenoid valves perform the functions within the orange dashed box in Fig 4.

The four solenoid valves were controlled by programs (available as online *Supporting Information*) written in *OCW*, a simple language we developed for controlling microfluidic valves [43]. Pneumatic connections between the four solenoid valves, the pneumatic RAM, and the soft robotic fingers were made using flexible laboratory tubing (1/16” ID, 1/8” OD) as shown in Fig 11.

### Characterizing pneumatic “memory”

A camera was used to record videos of a soft robotic finger while the pneumatic RAM trapped a vacuum inside the finger. The side view of the finger (shown in Fig 9A) and two white marks added to the finger facilitated the measurement of the finger’s deflection angle over time using a custom MATLAB script (provided as online *Supplementary Information*). The pneumatic RAM chip continued to contract and extend other fingers while the test finger was held contracted by the trapped vacuum “memory.” This test was repeated for a total of three trials with a different actuator each time (all results in online *Supplementary Information*); the trial shown in Fig 9B had the median trapped vacuum duration of the three trials.

### Playing notes and chords on a piano keyboard

A camera was used to record videos of several soft robotic fingers while playing chords (Fig 10) and songs (Fig 11) on a piano keyboard. The sound recording accompanying the video was used to determine the note-to-note delay times in Fig 10. The video recording of the soft robotic fingers playing “Mary Had a Little Lamb” (Fig 11) is available as online *Supplementary Information*.

## Supporting information

S1 File Design of the pneumatic RAM chip in Adobe Illustrator format. (AI)

S2 File Table showing the state of each valve in each step during the operation of the pneumatic RAM in Fig 6. (PDF)

S3 File Design of the 3D-printed soft robotic finger in SolidWorks format. (SLDPRT)

S4 File Code for measuring finger deflection angles in pneumatic memory experiments in MATLAB. (M)

S5 File Source code for the OCW programs written to control pneumatic integrated circuits. (ZIP)

S1 Video Video recording of a pneumatic RAM showing valve states during operation. (MP4)

S2 Video Video containing detailed frames of all 256 states of the eight soft robotic fingers from Fig 8. (MP4)

S3 Video Video recording of “Mary Had a Little Lamb” performed by a soft robot controlled by a pneumatic RAM chip. (MP4)

S1 Fig Closeup of the first few seconds of Fig 9. (PDF)

S2 Fig Additional results from pneumatic memory characterization experiments like the one in Fig 9. (PDF)
