# Supplementary material for: A pneumatic random-access memory for controlling soft robots
Source: PLoS One. 2021 Jul 16;16(7):e0254524. doi: 10.1371/journal.pone.0254524 (PMC8284813; doi:10.1371/journal.pone.0254524)
Supplement: S2 File — (PDF) [file pone.0254524.s002.pdf]

State of each valve in each step during the operation of the pneumatic demultiplexer IC in Figure 6 of the main text

Supplementary information for "A pneumatic random-access memory for controlling soft robots"
by Shane Hoang, Konstantinos Karydis, Philip Brisk, and William H. Grover, PLOS ONE

Step 1			
Valve	Receives:	From:	Valve State:
A	Atmosphere	Select Bit 0	Closed (VAA)
B	Vacuum	¬Select Bit 0	Open, then Closed (VVA -> VVV)
C	Vacuum	Select Bit 1	Open (AVA) *
D	Vacuum	Select Bit 1	Open, then Closed (VVA -> VVV)
E	Atmosphere	¬Select Bit 1	Closed (AAA)
F	Atmosphere	¬Select Bit 1	Closed (VAA)
G	Atmosphere	Select Bit 2	Closed (AAA)
H	Atmosphere	Select Bit 2	Closed (VAA)
I	Atmosphere	Select Bit 2	Closed (AAA)
J	Atmosphere	Select Bit 2	Closed (AAA)
K	Vacuum	¬Select Bit 2	Open (AVA) *
L	Vacuum	¬Select Bit 2	Open, then Closed (VVA -> VVV)
M	Vacuum	¬Select Bit 2	Open (AVA) *
N	Vacuum	¬Select Bit 2	Open (AVA) *

Step 2			
Valve	Receives:	From:	Valve State:
A	Atmosphere	Select Bit 0	Closed (VAA)
B	Vacuum	¬Select Bit 0	Open, then Closed (VVA -> VVV)
C	Vacuum	Select Bit 1	Open (AVA) *
D	Vacuum	Select Bit 1	Open, then Closed (VVA -> VVV)
E	Atmosphere	¬Select Bit 1	Closed (AAA)
F	Atmosphere	¬Select Bit 1	Closed (VAA)
G	Vacuum	Select Bit 2	Open (AVA) *
H	Vacuum	Select Bit 2	Open, then Closed (VVA -> VVV)
I	Vacuum	Select Bit 2	Open (AVA) *
J	Vacuum	Select Bit 2	Open (AVA) *
K	Atmosphere	¬Select Bit 2	Closed (AAA)
L	Atmosphere	¬Select Bit 2	Closed (VAV)
M	Atmosphere	¬Select Bit 2	Closed (AAA)
N	Atmosphere	¬Select Bit 2	Closed (AAA)

Step 3			
Valve	Receives:	From:	Valve State:
A	Vacuum	Select Bit 0	Open, then Closed (VVA -> VVV)
B	Atmosphere	¬Select Bit 0	Closed (VAA)
C	Atmosphere	Select Bit 1	Closed (VAA)
D	Atmosphere	Select Bit 1	Closed (AAV)
E	Vacuum	¬Select Bit 1	Open, then Closed (VVA -> VVV)
F	Vacuum	¬Select Bit 1	Open (AVA) *
G	Atmosphere	Select Bit 2	Closed (AAA)
H	Atmosphere	Select Bit 2	Closed (VAV)
I	Atmosphere	Select Bit 2	Closed (VAA)
J	Atmosphere	Select Bit 2	Closed (AAA)
K	Vacuum	¬Select Bit 2	Open (AVA) *
L	Vacuum	¬Select Bit 2	Closed (VVV)
M	Vacuum	¬Select Bit 2	Open, then Closed (VVA -> VVV)
N	Vacuum	¬Select Bit 2	Open (AVA) *

Step 4			
Valve	Receives:	From:	Valve State:
A	Atmosphere	Select Bit 0	Closed (AAA)
B	Vacuum	¬Select Bit 0	Open (AVA)
C	Vacuum	Select Bit 1	Open (AVA) *
D	Vacuum	Select Bit 1	Open (AVA)
E	Atmosphere	¬Select Bit 1	Closed (AAV)
F	Atmosphere	¬Select Bit 1	Closed (AAA)
G	Atmosphere	Select Bit 2	Closed (AAA)
H	Atmosphere	Select Bit 2	Closed (AAV)
I	Atmosphere	Select Bit 2	Closed (VAA)
J	Atmosphere	Select Bit 2	Closed (AAA)
K	Vacuum	¬Select Bit 2	Open (AVA) *
L	Vacuum	¬Select Bit 2	Open (AVA)
M	Vacuum	¬Select Bit 2	Closed (VVV)
N	Vacuum	¬Select Bit 2	Open (AVA) *

* - Even though valve is open, other closed valves block flow through this valve during this step.
