# Supplementary material for: A pneumatic random-access memory for controlling soft robots
Source: PLoS One. 2021 Jul 16;16(7):e0254524. doi: 10.1371/journal.pone.0254524 (PMC8284813; doi:10.1371/journal.pone.0254524)
Supplement: S1 Fig — (PDF) [file pone.0254524.s009.pdf]

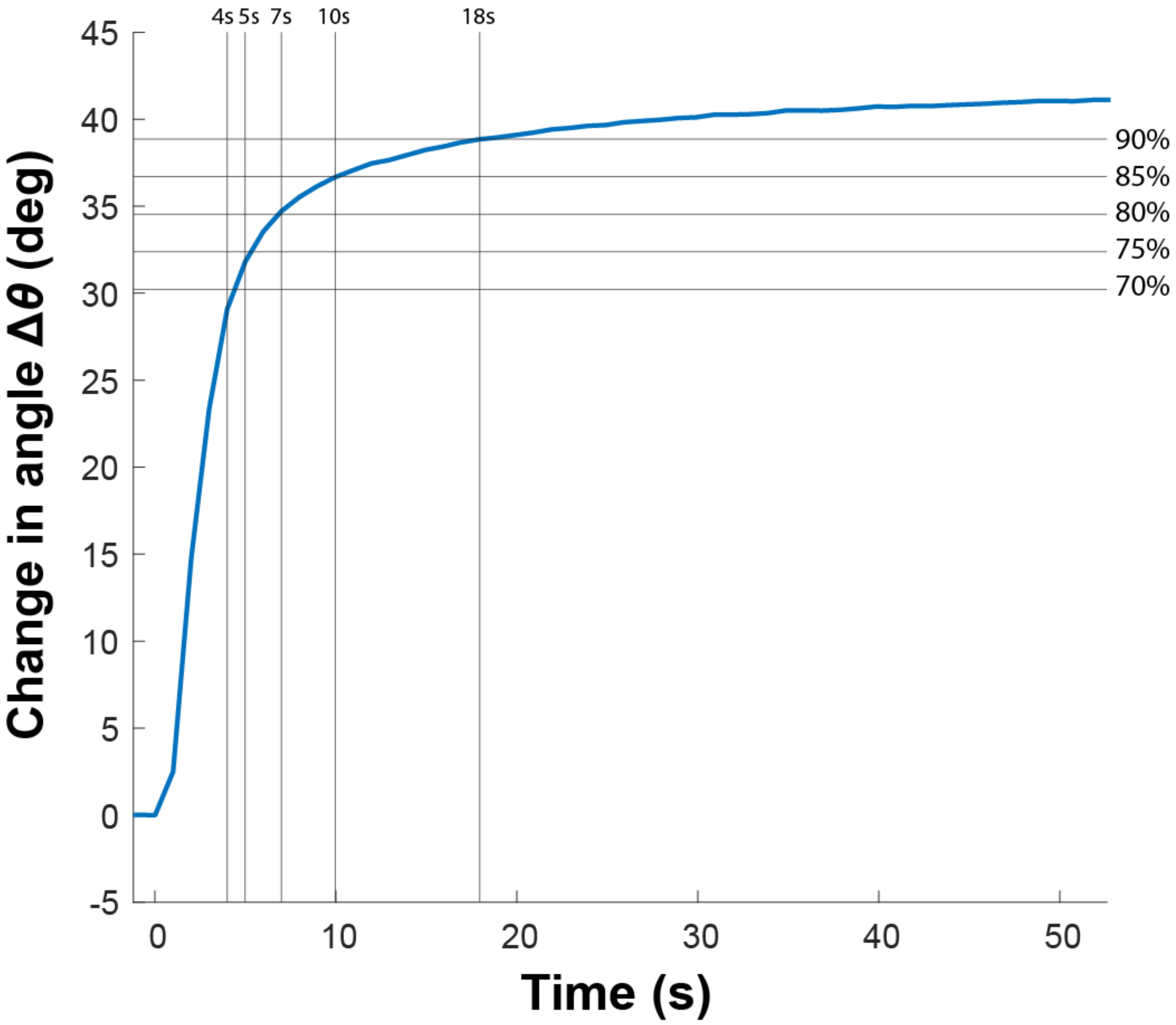

S1 Figure: Closeup of the first 50 seconds of Figure 9B from the main text. Controlled by the pneumatic demultiplexer IC, this soft robotic finger is 70% contracted after 4 seconds of applied vacuum, 75% contracted after 5 s, 80% contracted after 7 s, 85% contracted after 10 s, and 90% contracted after 18 s.
