# Supplementary material for: A pneumatic random-access memory for controlling soft robots
Source: PLoS One. 2021 Jul 16;16(7):e0254524. doi: 10.1371/journal.pone.0254524 (PMC8284813; doi:10.1371/journal.pone.0254524)
Supplement: S2 Fig — (PDF) [file pone.0254524.s010.pdf]

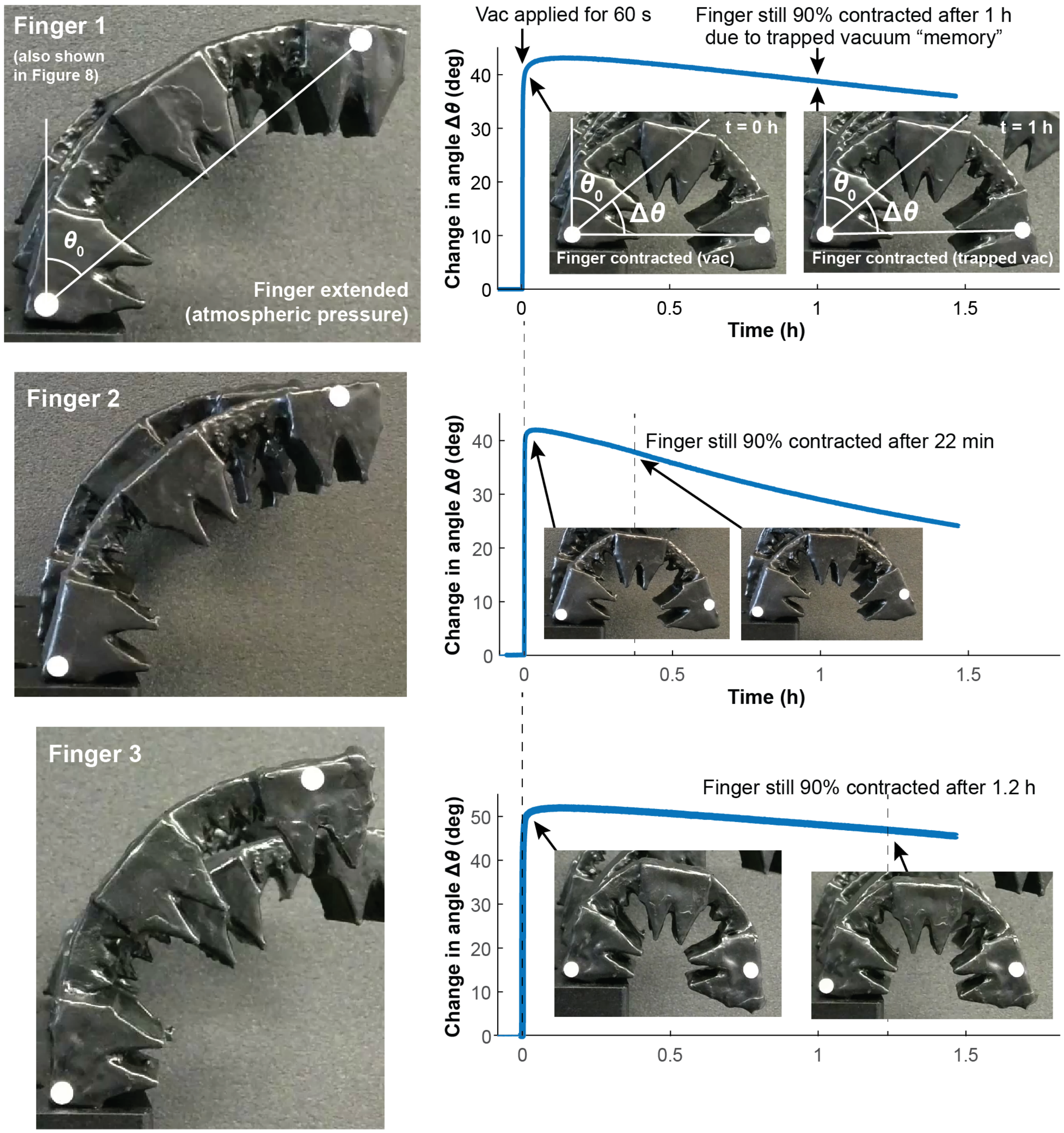

S2 Figure: Additional results from pneumatic memory characterization experiments. Finger 1 was also shown in the main text in Figure 9. The vacuum trapped by the pneumatic demultiplexer IC keeps a soft robotic finger at least 90% contracted for durations ranging from 22 minutes (Finger 2) to 1.2 hours (Finger 3).
